# Central coordination as an alternative for local coordination in a multicenter randomized controlled trial: the FAITH trial experience

**DOI:** 10.1186/1745-6215-13-5

**Published:** 2012-01-08

**Authors:** Stephanie M Zielinski, Helena Viveiros, Martin J Heetveld, Marc F Swiontkowski, Mohit Bhandari, Peter Patka, Esther MM Van Lieshout

**Affiliations:** 1 Dept. of Surgery-Traumatology, Erasmus MC, University Medical Center Rotterdam, P.O. Box 2040, 3000 CA Rotterdam, the Netherlands; 2 Dept. of Clinical Epidemiology and Biostatistics, McMaster University, HSC 2C, 1200 Main Street West, Hamilton, ON, L8N 3Z5, Canada; 3 Dept. of Surgery, Kennemer Gasthuis, P.O. Box 417, 2000 AK, Haarlem, the Netherlands; 4 Dept. of Orthopaedic Surgery, University of Minnesota Medical School, 2512 South 7th Street, Suite R200, Minneapolis, MN, 55454, USA; 5 Dept. of Emergency Medicine, Erasmus MC, University Medical Center Rotterdam, P.O. Box 2010, 3000 CA Rotterdam, the Netherlands

**Keywords:** randomized controlled trial, management, trial coordinator, trial performance, inclusion, follow-up

## Abstract

**Background:**

Surgeons in the Netherlands, Canada and the US participate in the FAITH trial (Fixation using Alternative Implants for the Treatment of Hip fractures). Dutch sites are managed and visited by a financed central trial coordinator, whereas most Canadian and US sites have local study coordinators and receive per patient payment. This study was aimed to assess how these different trial management strategies affected trial performance.

**Methods:**

Details related to obtaining ethics approval, time to trial start-up, inclusion, and percentage completed follow-ups were collected for each trial site and compared. Pre-trial screening data were compared with actual inclusion rates.

**Results:**

Median trial start-up ranged from 41 days (P25-P75 10-139) in the Netherlands to 232 days (P25-P75 98-423) in Canada (p = 0.027). The inclusion rate was highest in the Netherlands; median 1.03 patients (P25-P75 0.43-2.21) per site per month, representing 34.4% of the total eligible population. It was lowest in Canada; 0.14 inclusions (P25-P75 0.00-0.28), representing 3.9% of eligible patients (p < 0.001). The percentage completed follow-ups was 83% for Canadian and Dutch sites and 70% for US sites (p = 0.217).

**Conclusions:**

In this trial, a central financed trial coordinator to manage all trial related tasks in participating sites resulted in better trial progression and a similar follow-up. It is therefore a suitable alternative for appointing these tasks to local research assistants. The central coordinator approach can enable smaller regional hospitals to participate in multicenter randomized controlled trials. Circumstances such as available budget, sample size, and geographical area should however be taken into account when choosing a management strategy.

**Trial Registration:**

ClinicalTrials.gov: NCT00761813

## Background

Randomized controlled trials (RCTs) are generally perceived as the reference standard for generating valid scientific evidence on the evaluation of medical treatments and interventions [1]. Unfortunately, RCTs continue to be relatively scarce in the orthopedic trauma literature [2]. This may be attributed to the logistical challenges of the implementation of RCTs.

One of the most apparent challenges of RCTs is to recruit the required number of patients as timely and efficiently as possible [3]. Availability of fewer patients than expected usually leads to an extended trial period and increased costs. Multicenter collaborations offer the potential of recruiting more patients within a shorter time period, which can be advantageous if large patient numbers are required or if the targeted population has a low incidence. They also have the advantage of increased generalizability of the results [4,5]. However, the conduct of multicenter clinical trials requires a complex organization, which applies even more to international trials [6,7].

Obtaining ethics or Institutional Review Board (IRB) approval is a potential cause of delay, as procedures, documents, and legislation may vary between countries [8]. This process is often time-consuming and it is recommended to have dedicated and well-trained study personnel available to assist participating sites with these administrative tasks [9-13].

Another challenge in multicenter research is the selection and recruitment of appropriate participating clinical sites. Every participating site should have a devoted and dedicated clinician as site principal investigator. As surgeons often lack the time to spend on research, it is important to have an assisting research team that can adopt many of the time consuming research tasks. Having adequate support can be more important for sites to decide to participate, than offering a financial compensation for participation [14,15]. The presence of a trial coordinator or assistant will also facilitate an appropriate study infrastructure, which is a requirement for proper study conduct [3,8,16-18]. As community hospitals generally lack such infrastructure, they often cannot participate in multicenter trials. This is unfortunate, as some injuries or interventions are much more frequent in community hospitals than in university hospitals. Their participation in a multicenter trial could therefore positively influence the recruitment rate. Once a large multicenter or multinational RCT has started, a relatively complex organization should be implemented and maintained in order to assure a complete patient follow-up and a high quality in data management [19,20].

Usually individual sites are required to manage their local ethics procedures and trial logistics. They generally receive per patient payments as compensation. As an alternative option, a single trial coordinator can be appointed to manage all trial-related tasks for multiple sites in a certain geographic area or country, in which travel time is limited and does not include air fares. This is dependent on availability of full financial support, but can relieve local sites from many trial related tasks. This may attract more sites to participate, provide a smoother process of obtaining ethics approval, quicker trial start-up, higher inclusion rates and follow-up completeness.

Similar management strategies have been applied in the FAITH trial (Fixation using Alternative Implants for the Treatment of Hip fractures, NCT00761813). The FAITH trial is an international multicenter study initiated by the IHFRC (International Hip Fracture Research Collaborative) [21]. The primary objective of this trial is to assess the impact of sliding hip screws versus cancellous screw fixation on rates of revision surgery at two years in elderly patients with femoral neck fractures. This trial has been launched in over 60 sites, predominantly situated in the Netherlands, Canada, and the US. All sites in the Netherlands are managed and visited by a single financed national trial coordinator. Most Canadian and US sites have individual local site coordinators and receive per patient payments. The aim of this study was to assess how these strategies affected performance of the FAITH trial.

## Methods

### Study Characteristics

In the Netherlands 14 hospitals participated in the FAITH trial. In Canada 11 hospitals participated, and in the US 29. Besides patient enrolment, sites were also required to register all patients that were excluded or missed for inclusion.

In the period before the trial started 26 of the participating Dutch, Canadian and US sites started prospective screening for patients during a short defined period to explore the amount and rate of potential inclusions that could be expected from each site. The results of this prospective screening study were used for the further planning of the definitive trial.

Two different strategies were applied for the organization and management of the FAITH trial in these participating countries. In the Netherlands the trial was coordinated and managed from a university hospital. One central, national trial coordinator was appointed upon obtaining adequate funding. This was a medical doctor working on her PhD project. This coordinator was responsible for all study related tasks at all fourteen participating sites. She arranged the process of ethics approval and the necessary documents for all sites, initiated study start-up, maintained communication with sites and the methods center, randomized all patients, and collected all follow-up data for 250 patients. During a period when study related tasks became too much to handle for a single person, she was assisted by the research team at the central coordinating site. There was no local research support at the participating sites. The coordinator travelled regularly to all participating sites, which were within a range of maximum 114 kilometers from the coordinating site. Participating sites were only responsible for patient selection. Sites received no payment; all funding was used for covering costs of the central trial coordination.

Most Canadian and US sites (located in various states ranging from Nova Scotia to British Columbia and from California to New York) were responsible themselves for all local study related tasks. The vast majority of these sites have individual, local, research assistants that take care of these tasks for the FAITH trial, as well as for other trials in that hospital. As compensation these sites receive per patient payment. In these countries there was no central, national trial coordinator.

All countries were supervised by the FAITH trial central methods center and steering committee. In order to monitor progress and to keep participating sites focused, there was contact between the methods center and the Dutch central coordinator, Canadian and US local principal investigators on a weekly base. The Dutch central coordinator had weekly contact with the participating sites and all sites received monthly newsletters showing the progress of the trial.

### Data

Data related to trial initiation, organization, and performance were collected up to August 11, 2010. At this time patient recruitment was still ongoing in Canada and the US. Data were collected concerning:

- baseline characteristics; country, type of hospital (*i.e*., university, non-university teaching, or non-university non-teaching), type of research coordinator (*i.e*., not available, site-specific or provided for by central coordinating site);

- process of obtaining ethics approval; submission and approval date, number of submissions and type of revisions (*i.e*., changes in wording or content of the informed consent form, changes in in- or exclusion criteria, changes in the wording or content of the study protocol, extra information on the study protocol and procedures, extra information on financial aspects, or request for additional documents);

- pre-trial screening period; screening start and stop date, total number of patients screened and number of eligible patients screened;

- trial period: trial start and (if applicable) stop dates, total number of included, excluded and missed patients that were registered, total number of patients that were missed for registration (for the Netherlands only), total amount of kilometers travelled by research coordinator and associated costs (for the Netherlands only), follow-up completeness.

Additional variables that were calculated from these data are described in Table 1.

**Table 1:** Additional variables calculated concerning the period of obtaining ethics approval and the screening and trial period

Variable	Calculation
Time necessary for ethics/IRB approval (days)	a - b
Time between ethics/IRB approval and start trial (days)	c - a
Screening period (days)	f - e
Enrolment/trial period (days)	d - c
Total number of patients in screening period (n per month)	g/(f - e)
Number of eligible patients in screening period (n per month)	h/(f - e)
Proportion eligible patients of total in screening period (%)	(h/g) * 100
Total number of patients per month in trial period (n per month)	(i + j)/(d - c)
Number of inclusions in trial period (n per month)	i/(d - c)
Proportion inclusions of total in trial period (%)	(i/(i + j)) * 100
Proportion patients that were missed for registration in trial period of total (%)	(k/(i + j + k)) * 100
Rate of total number of patients per month in trial period versus screening period	((i + j)/(d - c))/(g/(f - e))
Rate of number of inclusions/eligible patients per month in trial period versus screening period	(i/(d - c))/(h/(f - e))
Rate of percentage inclusions/eligible patients per month in trial period versus screening period	((i/(i + j) * 100)/((h/g) * 100)

a, ethics/IRB approval date; b, ethics/IRB submission date; c, trial start date; d, trial stop date; e, screening start date; f, screening stop date; g, total number of patients screened; h, number of eligible patients screened; i, number of inclusions; j, number of excluded or missed patients that were registered; k, number of patients that were missed for registration.

### Data Analysis

All analyses were conducted using SPSS (version 16.0, SPSS Inc., Chicago, IL, USA). Data from the three countries (the Netherlands, Canada and US) were compared. The choice to compare these three countries was made because of the differences in trial management between the Netherlands and Canada/US described above. Comparing these countries separately also allowed the possibility to study country related differences that may affect trial performance, independent from the trial management strategy chosen. Continuous variables are presented as median with interquartile ranges. Categorical variables are presented as number (percentage). Continuous variables were compared with the Kruskal-Wallis Analysis of Variance (ANOVA). Post-hoc pair wise comparisons were performed using the Mann-Whitney U-test. Categorical variables were compared with the Chi-squared test. A P-value < 0.05 (two-sided) was taken as threshold of statistical significance.

## Results

### Characteristics of participating sites

The type of hospitals participating was similar for the Netherlands (NL), Canada (CA) and the US (Table 2). All sites in the Netherlands were centrally coordinated, whereas local site coordination was available at 72.7% of Canadian sites and 96.6% of US sites.

**Table 2:** Characteristics of countries participating in the FAITH trial

	NL (N = 14)	CA (N = 11)	US (N = 29)	P-value
Type of hospital				
University	4 (28.6)	9 (81.8)	16 (55.2)	0.051
Non-university teaching	10 (71.4)	2 (18.2)	11 (37.9)	
Non-university non-teaching	0 (0.0)	0 (0.0)	2 (6.9)	
Trial coordinator				
Not available	0 (0.0)	0 (0.0)	1 (3.4)	< 0.001
Available at site	0 (0.0)	8 (72.7)	28 (96.6)	
Provided by central coordinating site	14 (100.0)	3 (27.3)	0 (0.0)	

NL, the Netherlands; CA, Canada; US, United States.

Numbers in the headers represent the number of sites participating per country.

Data are presented as numbers with percentage between brackets. Statistics were calculated using the Chi-squared test.

### Process of obtaining ethics/IRB approval

The time necessary for ethics/IRB approval was significantly longer in the Netherlands (median 104 days) than in Canada (median 55 days) and the US (median 53 days; p = 0.027; Table 3). For all countries the median number of submissions requested by the ethics committee was one. However, due to the differences in data distribution and skewness there was still a significant difference in requested submissions between NL (P25-P75 0.0-1.0) and the US (P25-P75 1.0-3.0) (p = 0.014). The type of revisions requested did not differ between the countries; the vast majority concerned wording and content of the informed consent form.

**Table 3:** Data concerning the process of obtaining ethics/IRB approval of countries participating in the FAITH trial

	NL (N = 14)	CA (N = 11)	US (N = 19)	P-value
Time necessary for ethics/IRB approval^1 ^(days)	104 (74-135)	55 (27 - 77)	53 (44 - 105)	0.027^+ a^
Revision rounds^1^	1 (0.0 - 1.0)	1 (0.8 - 1.0)	1 (1.0 - 3.0)	0.014^+ b^
Type of revisions requested:				
Wording of IC Form^2^	6 (42.9)	5 (50.0)	12 (66.7)	0.382^++^
Content of IC Form^2^	6 (42.9)	5 (50.0)	8 (44.4)	0.938^++^
In- or exclusion criteria^2^	0 (0.0)	1 (10.0)	1 (5.3)	0.511^++^
Wording of study protocol^2^	0 (0.0)	1 (10.0)	0 (0.0)	0.185^++^
Content of study protocol^2^	1 (7.1)	2 (20.0)	1 (5.3)	0.406^++^
Additional information in study protocol/procedures^2^	5 (35.7)	3 (30.0)	7 (36.8)	0.932^++^
Financial aspects - Request for extra information^2^	0 (0.0)	1 (10.0)	3 (15.8)	0.303^++^
Request additional documents^2^	2 (14.3)	0 (0.0)	3 (15.8)	0.421^++^

NL, the Netherlands; CA, Canada; US, United States.

Numbers in the headers represent the number of sites per country for which data were available.

IC, informed consent form.

^1 ^Data are presented as median with P_25_-P_75 _given between brackets. ^2 ^Data are presented as number with percentages.

^+ ^Kruskal-Wallis ANOVA, ^++ ^Chi-squared test

Post-hoc pair wise comparisons were performed using the Mann-Whitney U-test: ^a ^Statistical significance was reached when comparing NL vs. CA (p = 0.025), and NL vs. US (p = 0.019), CA vs. US: not significant. ^b ^Statistical significance was reached when comparing NL vs. US (p = 0.007), other groups: not significant

### Pre-trial screening period

Of the currently participating FAITH sites four Dutch, eight Canadian, and fourteen US sites also took part in the pre-trial prospective screening period. The duration of the screening period did not differ significantly between groups (Table 4). The number of patients screened per site was least in the Netherlands with six patients in total and 3.3 patients per month. Forecasted total number of patients was highest for Canadian sites with 15 patients per site in total and 7.5 per month (p = 0.006 and p = 0.016). Other variables, concerning the amount and proportion of eligible patients screened, were not significantly different between countries.

**Table 4:** Data concerning the pre-trial screening period of countries participating in the FAITH trial

	NL (N = 4)	CA (N = 8)	US (N = 14)	P-value
Screening period (days)	55 (51 - 92)	60 (56 - 74)	50 (20 - 69)	0.121
Eligible patients (N)	4 (3 - 6)	4 (1 - 10)	3 (1 - 6)	0.571
Total patients (N)	6 (6 - 9)	15 (15 - 34)	6 (5 - 11)	0.006^a^
Eligible patients (N per month)	2.0 (1.7 - 2.4)	1.5 (0.7 - 4.1)	2.2 (1.1 - 5.0)	0.786
Total patients (N per month)	3.3 (3.0 - 3.6)	7.5 (5.6 - 17.4)	5.0 (3.1 - 8.2)	0.016 ^b^
Proportion eligible patients (% of total)	63 (53 - 67)	14 (10 - 40)	48 (29 - 71)	0.062

NL, the Netherlands; CA, Canada; US, United States.

Numbers in the headers represent the number of sites per country that data were available for.

Data are presented as median with P_25_-P_75 _given between brackets.

Statistics were calculated using the Kruskal-Wallis ANOVA.

Post-hoc pair wise comparisons were performed using Mann-Whitney U-test: ^a ^Statistical significance was reached when comparing NL vs. CA (p = 0.009), and CA vs. USA (p = 0.004), NL vs. USA: not significant. ^b ^Statistical significance was reached when comparing NL vs. CA (p = 0.007), other groups: not significant.

### Trial period

Dutch hospitals started enrolment in the period between February 2008 and October 2008. In August 2009 the national goal of enrolling 250 patients was achieved. Canadian hospitals started enrolment between March 2008 and June 2010. In the US hospitals started enrolment between February 2009 and September 2010. In Canada and the US enrolment is still ongoing.

**Figure 1:**
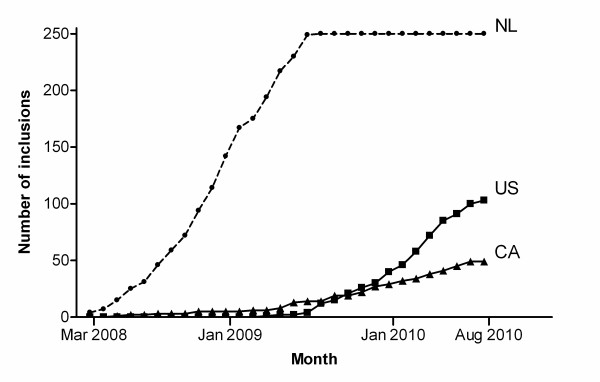
**Inclusion progression for the Netherlands (NL), Canada (CA) and the United States (US)**.

The time necessary for trial start-up was defined as the time between obtained ethics approval and the actual trial start up. With a median of 41 days, trial start-up was fastest for the Netherlands. Trial start-up took more than five times longer for Canada (median 232 days; p = 0.027; Table 5). Because the median enrolment period was statistically significantly longer for the US than for the Netherlands (median 283 vs. 423 days, respectively, p = 0.001), crude numbers were also calculated per month. The total number of patients seen per month in the trial period was similar in all countries; however, the inclusion rate in the Dutch sites per month was more than three times higher than in the US sites, and more than seven times higher compared with Canadian sites (1.03 patients per month vs. 0.31 and 0.14, respectively, p < 0.001). Inclusion progression of all countries is also shown in Figure 1. Similar differences were seen when comparing the proportion inclusions of the total patient group. In Dutch sites 34.4% of the patients were included vs. 16.7% in US sites and 3.93% in Canadian sites (p = 0.001). These numbers may however be influenced by the varying screening compliance in all countries. For example, sites screening all hip fractures would certainly have greater screening failure rates then those sites screening only those hip fractures that were deemed treatable by internal fixation.

**Table 5:** Data concerning the trial period of countries participating in the FAITH trial

	NL (N = 14)	CA (N = 11)	US (N = 29)	P-value
Time between ethics approval and start trial (days)	41 (10 - 139)	232 (98 - 423)	87 (45 - 255)	0.027^a^
Enrolment period (days)	423 (381 - 509)	482 (267 - 663)	283 (142 - 360)	0.001^b^
Inclusions	13 (7 - 27)	3 (0 - 5)	3 (1 - 6)	< 0.001^c^
Registered patients	23 (12 - 36)	54 (3 - 75)	16 (5 - 27)	0.060
Patients missed for registration^1^	35 (10 - 81)	Unknown	Unknown	
Inclusions (n per month)	1.03 (0.43 - 2.21)	0.14 (0.00 - 0.28)	0.31 (0.09 - 0.62)	< 0.001^d^
Total patients (n per month)	2.49 (1.60 - 3.64)	2.76 (0.60 - 8.75)	1.96 (1.11 - 3.75)	0.574
Proportion inclusions (% of total)	34.4 (23.8 - 62.6)	3.93 (0.00 - 13.2)	16.7 (2.50 - 31.3)	0.001^e^
Proportion patients that were missed for registration of total^1 ^(%)	57.4 (32.2 - 65.2)	Unknown	Unknown	
Completed follow-ups (%)	82.6 (80.0 - 84.6)	83.5 (72.7 - 95.2)	70.0 (60.0 - 88.1)	0.217
Follow-ups in window (%)	77.1 (71.0 - 82.2)	85.9 (81.0 - 95.0)	85.7 (70.0 - 100.0)	0.073

NL, the Netherlands; CA, Canada; US, United States.

Numbers in the headers represent the number of sites per country that data were available for.

Data are presented as median with P_25_-P_75 _given between brackets.

Statistics were calculated using the Kruskal-Wallis ANOVA.

^1 ^Data available for NL only.

Post-hoc pair wise comparisons were performed using a Mann-Whitney U-test: ^a ^Statistical significance was reached when comparing NL vs. CA (p = 0.010), other groups: not significant. ^b ^Statistical significance was reached when comparing NL vs. US (p < 0.001), other groups: not significant. ^c ^Statistical significance was reached when comparing NL vs. CA (p = 0.002) and NL vs. US (p < 0.001), CA vs. US: not significant. ^d ^Statistical significance was reached when comparing NL vs. CA (p = 0.001) and NL vs. US (p = 0.001), CA vs. US: not significant. ^e ^Statistical significance was reached when comparing NL vs. CA (p < 0.001) and NL vs. US (p = 0.009), CA vs. US: not significant.

For sites in the Netherlands data were extracted from hospital records in order to check for omissions in patient registrations during the trial. A median of 35 excluded and missed patients (P25-P75 10-81) were not registered per site, despite clear instructions to participating sites that this was required for the trial. This represented 57.4% of the total amount of patients seen during the trial period. These data were not available for the other countries.

Follow-up data were collected at eight time points, four times in clinic and four times by telephone. The percentage completed follow-ups were calculated for all time points and the overall percentage completed follow-ups was computed. The median overall percentage completed follow-ups was 70% in Canada, and exceeded 80% in the Netherlands and US. No statistically significant differences were found between countries (Table 5). The median percentage of follow-ups that were completed within the predefined acceptable time window was 77% in the Netherlands, and 86% in Canada and the US. Again, this was not statistically significantly different (Table 5).

During this study, the central trial coordinator in the Netherlands travelled 28,842 kilometers in order to visit all fourteen participating sites for trial start-up, enrolment, and data collection in clinic. This resulted in € 9,771 total travel costs.

### Pre-trial screening period versus trial period

**Figure 2:**
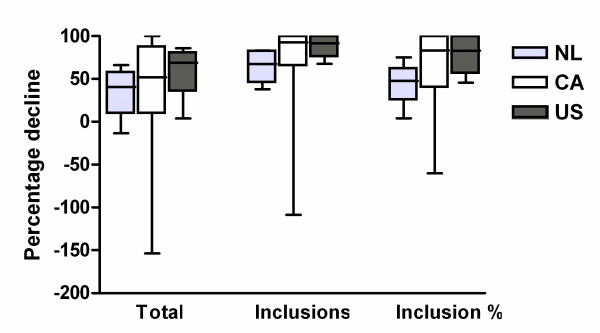
**The percentage decline in total number of patients seen per month, number of inclusions per month and percentage inclusions of total number of patients, during the trial period in comparison with the pre-trial screening period**. To calculate this percentage decline, the total number of patients seen in the trial period was divided by the total number of patients seen in the pre-screening trial period. Similar calculations were made for the number of inclusions per month and the inclusion percentage. These rates (a) were transformed to a percentage decline (b) using the following formula: b = (1-a) * 100%. This figure therefore shows that for all variables there were fewer patients during the trial period compared with the pre-trial screening period in all countries. NL, the Netherlands; CA, Canada; US, United States.

Pre-trial screening data regarding eligible patients were compared with the actual inclusion rates and percentages in the trial period, to study the value of pre-trial screening data. An overview of the calculated variables is shown in Table 1. Inclusion rates in the trial were much lower than expected from the screening period: a decline of 67% (P25-P75 42-83) was noted for sites in the Netherlands versus a decline of 92% (P25-P75 78-100) for US and 93% (P25-P75 68-100) for Canadian sites (p = 0.154; Figure 2). The total number of patients seen in the trial period versus the screening period also displayed a decline: 41% less (P25-P75 -2-62) for the Netherlands versus 52% (P25-P75 9-88) for Canada and 69% (P25-P75 35-81) for the US (p = 0.477). Consequently, the proportion inclusions of the total patient group also decreased: 48% decrease (P25-P75 14-69) for sites in the Netherlands versus 83% (P25-P75 39-100) for Canadian sites and 83% (P25-P75 56-100) for US sites (p = 0.091).

## Discussion

In this study, trial progression in the Netherlands, where a central trial coordinator managed most tasks for multiple centers in a restricted geographical area, was better than in Canada and the US, where local research assistants were appointed at individual sites. The central trial coordinator system was associated with a shorter trial start-up time, higher inclusion rate, and a higher inclusion percentage. Collection of follow-up data was equally good in both systems.

The process of obtaining ethics approval can be time-consuming and stressful and may yield diverse responses from ethics committees [9-13]. For the FAITH trial, obtaining ethics approval took longer in the Netherlands than in Canada or the US. This may have been influenced by the time that the trial coordinator or sites needed to assemble forms for the ethics committee. Ethics submissions may have taken longer in the Netherlands at the very start of the trial, when there was no central trial coordinator yet. The longer approval process in the Netherlands may also have been caused by more inefficient medical ethics procedures [10]. In the Netherlands multicenter studies need approval from a central ethics committee that performs a full review of the study documents. Subsequently, ethics committees in participating sites should only advise on local feasibility. This two-step approach was aimed to simplify and shorten the ethics procedures for multicenter trials. It is nevertheless disputable if this goal is currently achieved [10]. In Canada and the US a single procedure for study review and approval is performed at all sites, which may turn out to be more efficient.

The number of resubmissions requested by the ethics committees was lower in the Netherlands, and can therefore not have contributed to the prolonged approval process. The availability of a central trial coordinator with detailed insight and knowledge on the study content could have prevented incorrect submissions in the Netherlands. In all countries remarks of the ethics committees mainly involved the informed consent form and extra information on the study protocol and procedures. Standardization of the ethics approval process is recommended as it may reduce the local differences in ethics approval terms.

The trial start-up period was statistically significantly shorter in the Netherlands (median 41 days) than in the US and Canada (87 and 232 days). In these latter two countries contract negotiations with participating sites had to take place during the trial start-up period, whereas this procedure was not applicable in the Netherlands. Furthermore, research assistants from Canada and the US frequently reported a long period between grant approval and official release of the funds. These aspects related to the per patient payment strategy applied in Canada and the US slowed the trial start-up process in these countries. In the Netherlands contract negotiations or per patient payments were unnecessary, as all tasks were performed by the central study coordinator, not resulting in delay. The assistance of the Dutch central trial coordinator in trial start-up activities (*e.g*. distributing study materials, giving start-up presentations) may also have contributed to a more efficient and speedy trial start-up.

Most influential to the differences in trial progression between the countries were the evident differences in inclusion rate and percentage, which were statistically significantly higher for sites in the Netherlands. The availability of the central trial coordinator in the Netherlands made it possible for smaller, non-university sites without a local research infrastructure or coordinator to participate. These sites generally treat more patients from the targeted population (*i.e*., femoral neck fracture patients) than the large university hospitals, but would normally not have been able or willing to participate in the trial, because they lack a local coordinator. Most of the principal investigators of these sites reported to be very motivated to enroll patients, as the (administrative) burden of participation was relatively low, thanks to the fact that the performance of follow-ups and other trial related tasks were adopted by the central trial coordinator. They declared that this was crucial in their decision to participate. This high devotion and lack of burdensome trial related tasks will probably have contributed to a good inclusion rate. Therefore, the availability of a central trial coordinator can contribute to a fast enrolment, both directly, by motivating local principal investigators to participate and enroll because of the low (administrative) burden of participation, and indirectly, by enabling high-volume hospitals without a local research network to participate. However, differences in accrual between countries may also have been affected by the known intercultural differences in preferred treatments for femoral neck fractures [22]. North American surgeons may have considered less femoral neck fracture patients suitable for internal fixation than Dutch surgeons do, as they are less committed to internal fixation as a preferred treatment. Moreover, surgeons from the US and Canada reported at investigator meetings that North American patients may be less lenient to participate in trials and that they experienced problems at obtaining informed consent [23]. Within the countries, higher accrual was also associated with a large target population, dedicated and compliant principal investigators, and low study related workload for participating surgeons. These are important aspects to pursue when planning a multicenter randomized controlled trial, and can be facilitated by appointing a central trial coordinator.

Comparison of the pre-trial prospective screening data with the actual trial data showed an obvious discrepancy in all countries. It is known that participating surgeons tend to overestimate enrolment numbers based upon a pre-trial screening period [24]. In this study accrual was also much lower than expected from the screening period. The use of pre-trial screening can therefore be debated. However, it may be useful to indicate good dedication to participate and help raise awareness for the upcoming study in potential sites. If a pre-trial screening is deemed necessary, a retrospective approach is recommended, as it is easier and results in similar estimated numbers, compared with a prospective approach [24].

The percentage completed follow-ups was not affected by the availability of a central trial coordinator and was between 70% and 84% for all countries. Follow-ups did seem to be completed within the window a bit less in the Netherlands, although not statistically significant. This was a result of the limitations that we experienced from the central trial coordinator approach. Usefulness of this approach will decrease with an increased sample size and an increased distance between sites. A single person can only manage a certain maximum number of sites and patients. Similarly, there is a maximum to the distance that can be traveled per day. In our study, a single study coordinator to manage 14 sites and 250 patients seemed somewhat limited. The study coordinator had to complete eight follow-ups per patient (four clinical and four telephonic), in 14 sites that were maximum 140 km. apart (maximum 2 hours travel time), resulting in an average of almost 1,000 km traveled per week. In these circumstances it was not always feasible to manage all follow-ups. We feel that it would have been optimal to have one coordinator following a maximum number of 200 patients, in our study. It is also important to have a supporting research team available for assistance if work pressure gets too high for a single person. The central coordinator approach is feasible within European countries, as well as within American/Canadian states.

Finally, a central coordinator may also contribute to the impartiality of the collected data and may prevent biases that could be introduced if the local coordinators/participating doctors are also the treating physician. Obviously, this could not be analyzed or proven in this study.

Obviously, this study has its limitations. Many of the results of this study were multifactorially influenced. Not all differences between countries can therefore be attributed to the difference in trial management system. If the two models (*i.e*., central management and local management) would have been conducted equally in each of the countries, bias due to country-specific conditions could have been ruled out. This was however impossible in the current study. Nevertheless, the availability of the central coordinator has certainly contributed to the speedier trial start-up, high enrolment rates and complete follow-up. Also, the limited number of sites available for data assemblage (especially for the screening period) and the fact that not all data were collected prospectively may have introduced some bias.

## Conclusions

In summary, trial performance can be influenced by the management strategy chosen. This study shows that the appointment of a central financed trial coordinator to manage all trial related tasks is a feasible alternative for the more traditional approach of appointing trial related tasks to local research assistants at participating sites. Taking important circumstances such as available budget, sample size, and geographical area into account, a central trial coordinator approach can add to the success of a multicenter randomized trial. A central coordinator should be considered when studying injuries that occur more frequently in smaller regional hospitals (without a local research coordinator). It will enable these sites to participate in randomized controlled trials, resulting in a enhanced enrolment rate. It should also be considered when the targeted principal investigators are unable to participate due to the (administrative) burden of participation. However, a central coordinator should only be considered for multiple sites in a restricted geographical area. Depending upon the geographic spread of the sites and the frequency of follow-up a careful estimation should be made of the amount of patients and sites that can be managed by a single coordinator.

## Competing interests

The authors declare that they have no competing interests.

## Authors' contributions

The authors made the following contributions:

S.Z. (Dutch coordinator FAITH trial): study design, data acquisition and analysis, interpretation of data, drafting and critical revision of the manuscript, approved final version

H.V. (Team member methods center FAITH trial): data acquisition and interpretation of data, critical revision of the manuscript, approved final version

M.H. (Steering committee member FAITH trial): study design, interpretation of data, drafting and critical revision of manuscript, approved final version

M.S. (Steering committee member FAITH trial): study design, interpretation of data, drafting and critical revision of manuscript, approved final version

M.B. (Steering committee chair FAITH trial): study design, interpretation of data, drafting and critical revision of manuscript, approved final version

P.P. (Site principal investigator FAITH trial): study design, interpretation of data, drafting and critical revision of manuscript, approved final version

E.V.L. (Research coordinator Erasmus MC): study design, data analysis and interpretation of data, drafting and critical revision of the manuscript, approved final version
